# Morel-Lavallée Lesion Treated With Single-Use Portable Negative Pressure Wound Therapy: A Case Report and Literature Review

**DOI:** 10.7759/cureus.78152

**Published:** 2025-01-28

**Authors:** Koshi Ota, Kazuaki Shimazu, Kanna Ota, Akira Takasu

**Affiliations:** 1 Department of Emergency and Critical Care Medicine, Osaka Medical and Pharmaceutical University, Takatsuki, JPN; 2 Department of Family Medicine, Osaka Healthcare Clinic, Osaka, JPN

**Keywords:** literature review, misdiagnosis, morel-lavallée lesion (mll), negative pressure wound therapy (npwt), trauma

## Abstract

A Morel-Lavallée lesion (MLL) is a traumatic, internal closed degloving injury that results from the shearing of the subcutaneous tissue away from the underlying fascia, usually associated with high-velocity trauma.

A 52-year-old man was transported to our hospital for further follow-up of a superficial femoral artery injury. Examination on admission showed bruising seen around the left thigh. Contrast-enhanced computed tomography of the left thigh revealed a hematoma with extravasation from a branch of the left superficial femoral artery, but no apparent aneurysms or femoral fractures. The patient was treated with compression bandages and discharged on hospital day 6. He left the hospital able to walk unaided. He subsequently complained of left thigh pain with swelling and visited the hospital several times. He was treated at another outpatient clinic and was diagnosed with MLL. Single-use disposable portable negative pressure wound therapy (NPWT) was applied for about two months, achieving a stable wound site.

We encountered a case of MLL successfully treated with single-use portable NPWT. This report presents the case of a patient with isolated MLL without fracture in the left thigh, in which the diagnosis was missed on initial admission. Finally, the patient received minor interventions including drainage along with tissue debridement and portable NPWT. We reviewed the relevant literature and summarized the pathogenesis, clinical manifestations, and treatment strategies for MLL. Emergency physicians require an increased understanding of MLL to avoid misdiagnosis and missed diagnosis.

## Introduction

A Morel-Lavallée lesion (MLL) is a traumatic, internal closed degloving injury that results when the subcutaneous tissue shears away from the underlying fascia, usually in association with high-velocity trauma [1-4]. Common causes of MLL are high-energy trauma, crush injury, or blunt force trauma [1-4]. Closed degloving occurs most frequently over the hip, flank, and proximal thigh, but can appear anywhere on the trunk and extremities [5]. Injury to areas with rich vascular and lymphatic supplies leads to the accumulation of hemolymphatic fluid (serosanguineous fluid) in this newly formed cavity generated by the separation of the superficial and deep fascia [2]. Necrotic material and blood products produce chronic inflammatory reactions. Over time, an encapsulated lesion lined by a fibrous capsule develops, filled with necrotic fatty tissue, blood products, debris, lymph, and fibrin, potentially leading to bacterial colonization and infection [4].

MLL typically presents within hours to days after the causative trauma. However, up to one-third of cases present months to years later and may be missed initially or difficult to associate with a specific inciting trauma event [5,6]. The clinical presentation of MLL varies widely from obvious ecchymosis, edema, and abrasions to the absence of any external signs. MLL can be confirmed by ultrasonography, computed tomography (CT), or magnetic resonance imaging (MRI) [5]. Early identification of MLL is essential because neglected lesions can become infected and may progress to extensive soft tissue necrosis.

The treatment for MLL is related to a variety of factors, such as lesion size, stage, and severity, but no guidelines for the management of MLL have yet been developed. Although many studies have reported the efficacy of various treatment regimens, such as conservative treatment, percutaneous aspiration, sclerotherapy, minimally invasive surgery, open surgery, or negative pressure wound therapy (NPWT), high-quality evidence remains lacking [5,7-10]. To the best of our knowledge, the number of cases of MLL treated with portable NPWT is very small, and no reviews have been reported.

Here, we describe the case of a middle-aged Japanese man with MLL who was successfully treated using portable NPWT. The aim of this study was to describe and evaluate the effectiveness and utility of using NPWT to treat MLL.

This article was previously posted to Preprints.org on June 18, 2024.

## Case presentation

**Figure 1:**
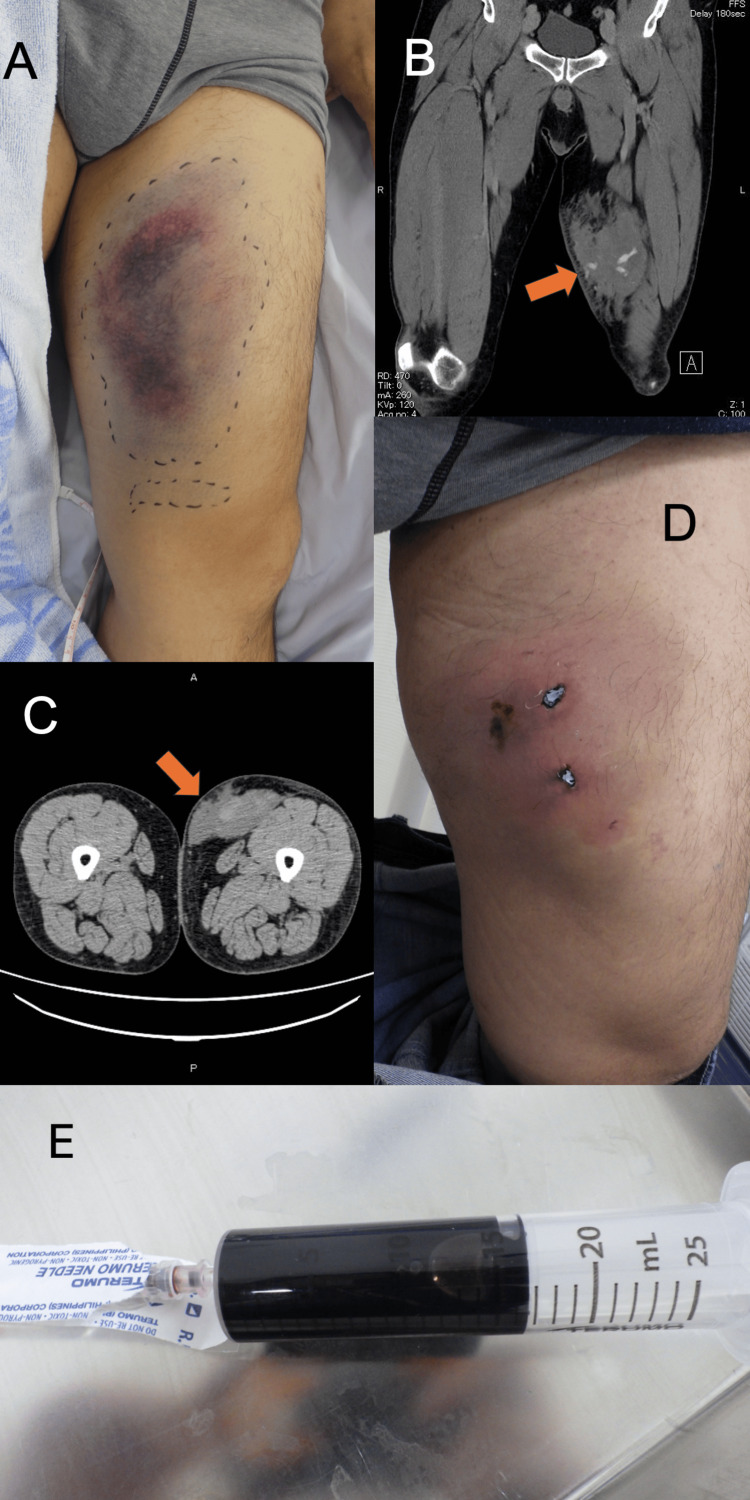
Left thigh Bruising and swelling are seen around the left thigh on the day of admission (A). Coronal contrast-enhanced CT shows a hematoma with extravasation of a branch of the left superficial femoral artery without apparent aneurysms or femoral fractures (B). Axial CT of the left thigh shows a subcutaneous hematoma in the left thigh (C). Orange arrows show the respective lesions. Bruising and swelling with two necrotic plaques are seen around the left thigh 10 days after discharge (D). Hematomatous fluid was drained by needle aspiration (E). Usually, serosanguineous fluid is drained by needle aspiration, but in this case, drainage was performed soon after injury; thus, hematomatous fluid was drained. CT: computed tomography

A 52-year-old Japanese man was transported to our hospital for further workup of a left thigh injury. He had fallen from a motorcycle after colliding with a car at a speed of around 30 km/h. He was initially transferred to another hospital, where contrast-enhanced CT showed a left thigh hematoma with extravasation from the superficial femoral artery. The patient was referred to our hospital for interventional radiology (IVR). He had a medical history of adjustment disorder with anxiety and had been prescribed sertraline hydrochloride and hydroxyzine hydrochloride once daily and clotiazepam as needed when he felt anxious. His habitual activities and family history were unremarkable. On arrival in the emergency room, his vital signs were as follows: temperature, 36.6°C; heart rate, 66 beats/min with regular rhythm; respiratory rate, 20 breaths/min; blood pressure, 118/94 mmHg; and oxygen saturation, 97% on room air. On examination, the patient was alert and complained of left thigh pain. Examination of the left thigh revealed bruising (Figure 1A). No other abnormalities were noted on examination of the left thigh. Physical examination of the chest, abdomen, and other limbs was unremarkable. Examination of cranial nerves showed no abnormalities. Contrast-enhanced CT of the left thigh revealed a hematoma with extravasation of a branch of the left superficial femoral artery, but no apparent aneurysms or fractures of the femur or pelvis (Figure 1B). IVR was therefore not indicated, and observational management with compression bandages was applied. The patient was admitted to our hospital for pain management and discharged on hospital day 6. He left the hospital able to walk unaided. The patient subsequently sought medical aid and visited our emergency department (ED) with left chest pain two days after discharge. There were no organic abnormalities found to account for the left-sided chest pain. It was attributed to psychological factors, and treatment with acetaminophen was initiated. Five days after discharge, he again visited our ED with the same left chest pain along with swelling in the left thigh and was again advised to use acetaminophen for pain. He revisited our ED 10 days after discharge complaining of pain, increased swelling, and loss of local sensation in the left thigh. CT of the thigh showed a subcutaneous hematoma in the left thigh with no evidence of fracture, with estimated dimensions of 10.9×5.55×12.5 cm (Figure 1C). Gross inspection 10 days after discharge revealed two necrotic plaques of roughly the same size with induration of the skin in surrounding areas (Figure 1D). Percutaneous aspiration was performed, and 15 mL of hematomatous fluid was drained by needle aspiration (Figure 1E) along with a total of 100 mL of clots after rupture of the two necrotic plaques.

**Figure 2:**
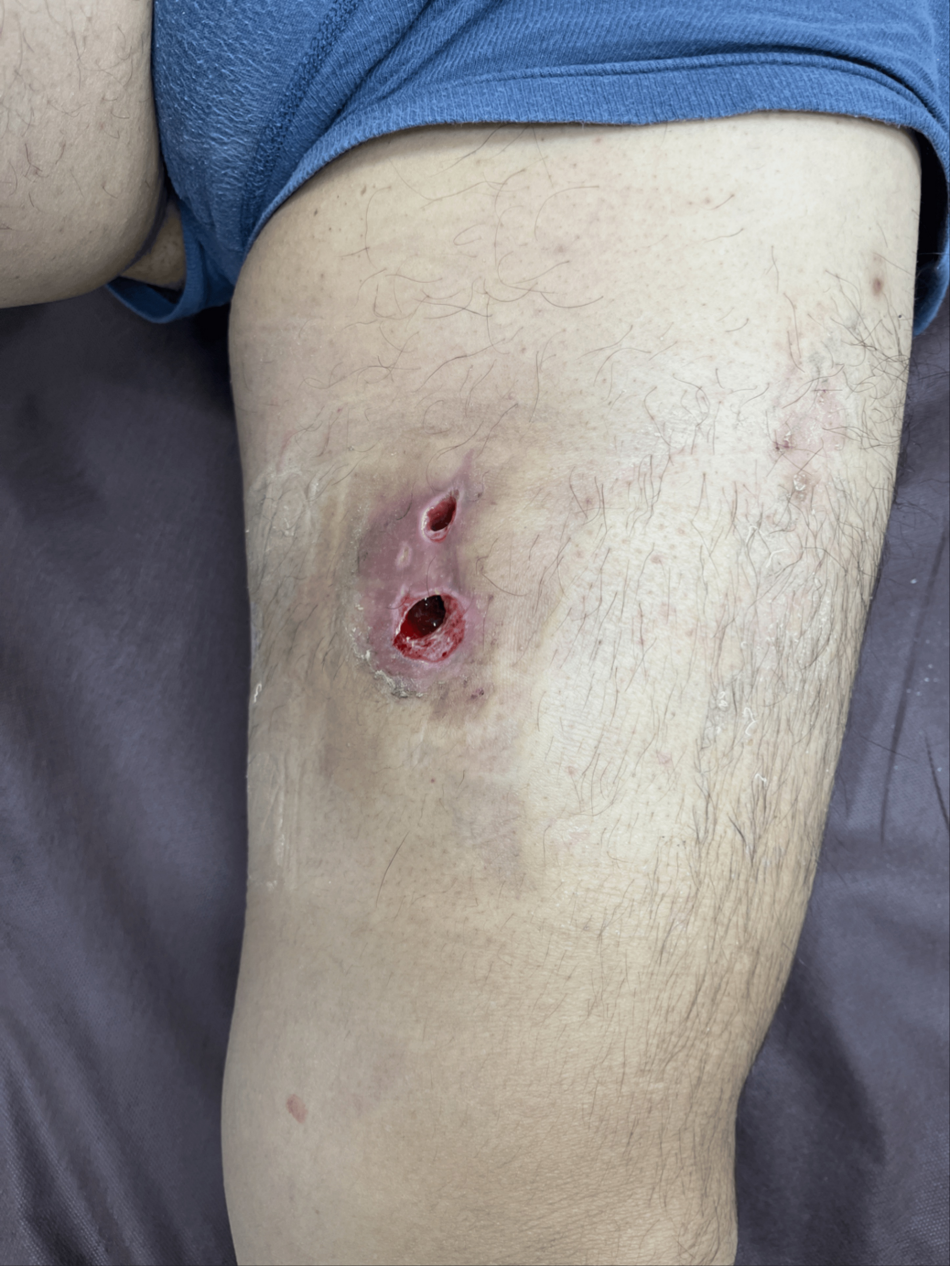
Treatment Two necrotic plaques were opened to extract clot with exudate, and we washed the cavity every 3-4 days.

Compression dressing was applied to the involved area using gauze and elastic bandages. A follow-up visit to another outpatient clinic was made 12 days after discharge, where wound care was provided. No necrotic tissue was observed, because follow-up treatment was provided relatively soon after the injury (Figure 2).

**Figure 3:**
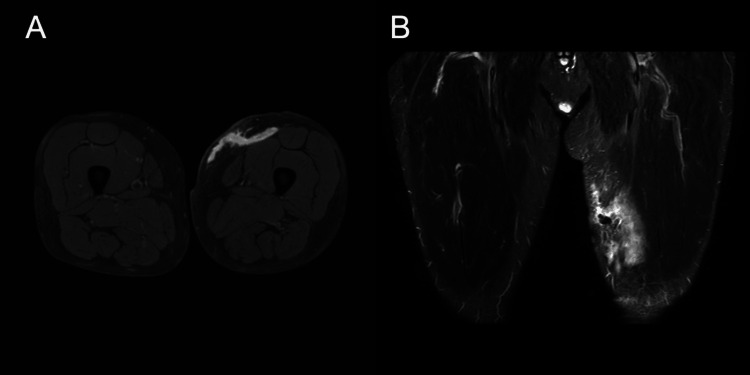
MRI on day 14 after discharge Axial T1 fat suppression MRI shows a hyperintense hematoma between the subdermal fat and fascia (A). Coronal short tau inversion recovery MRI shows a longitudinal, hyperintense hematoma about 100 mm in length between the subdermal fat and fascia (B). Hematoma was smaller than before because exudate and clots were extracted from two holes that had been necrotic plaques two days earlier in both A and B. MRI: magnetic resonance imaging

To confirm the diagnosis of MLL, an MRI of the left thigh was performed 14 days after discharge. T2-weighted MRI revealed cystic masses under the subcutaneous fat layer without a peripheral ring or capsule, as shown in Figure 3A, 3B.

**Figure 4:**
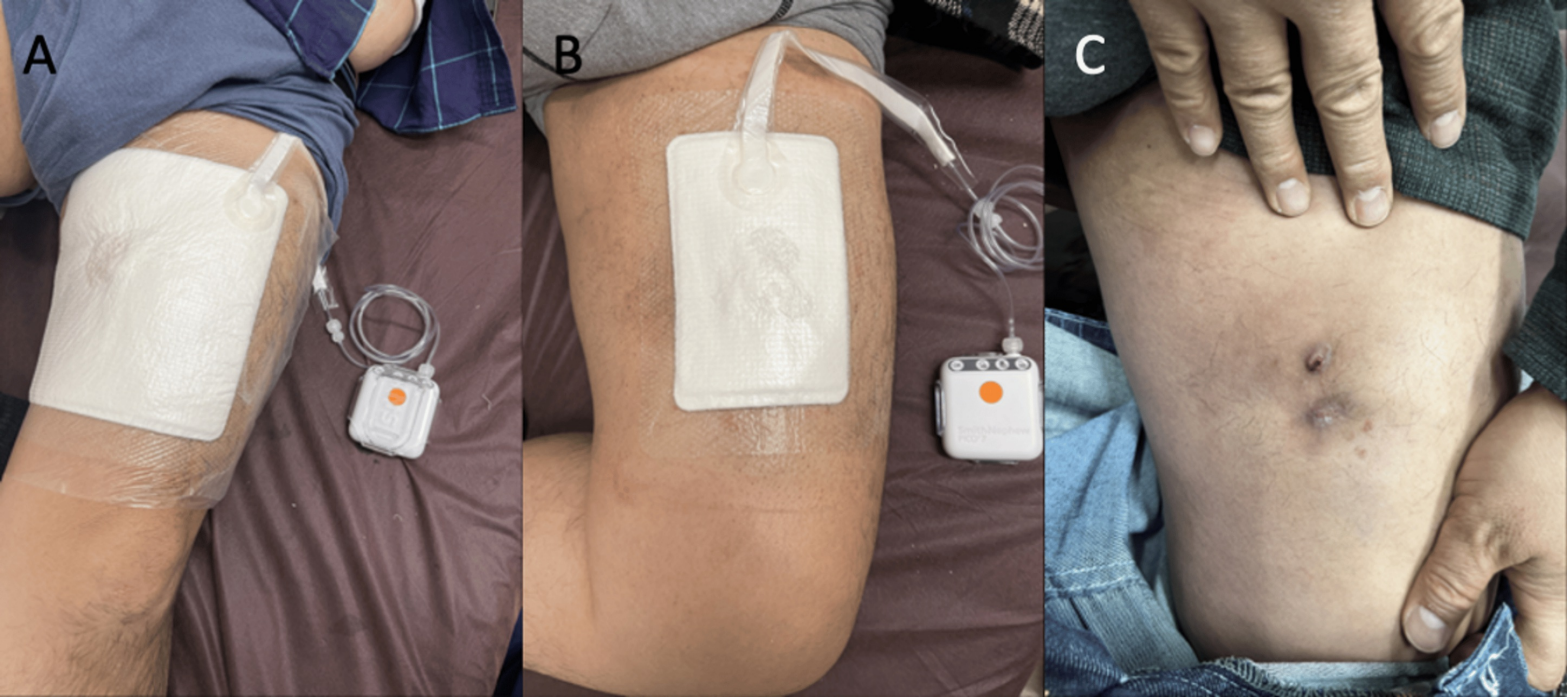
Treatment Single-use disposable portable NPWT was used on the two holes in the left thigh (PICO™; Smith & Nephew, London, UK). The pad was 25×25 cm in size (B). The cavity decreased in size, and the pad was then changed to 15×20 cm in size. Finally, the pad was changed to 15×15 cm in size (not shown). The wound was healed, but scarring and skin dent remained (D). NPWT: negative pressure wound therapy

Considering that the patient was ambulant, we changed the treatment plan and applied a single-use disposable portable NPWT system (PICO™; Smith & Nephew, London, UK) after several weeks of compression treatment (Figure 4A, 4B). NPWT dressings were changed with simultaneous serial debridement at 3-4-day intervals for 57 days. MLL completely resolved, but the patient felt discomfort and skin indentation over the site of MLL (Figure 4C).

## Discussion

**Figure 5:**
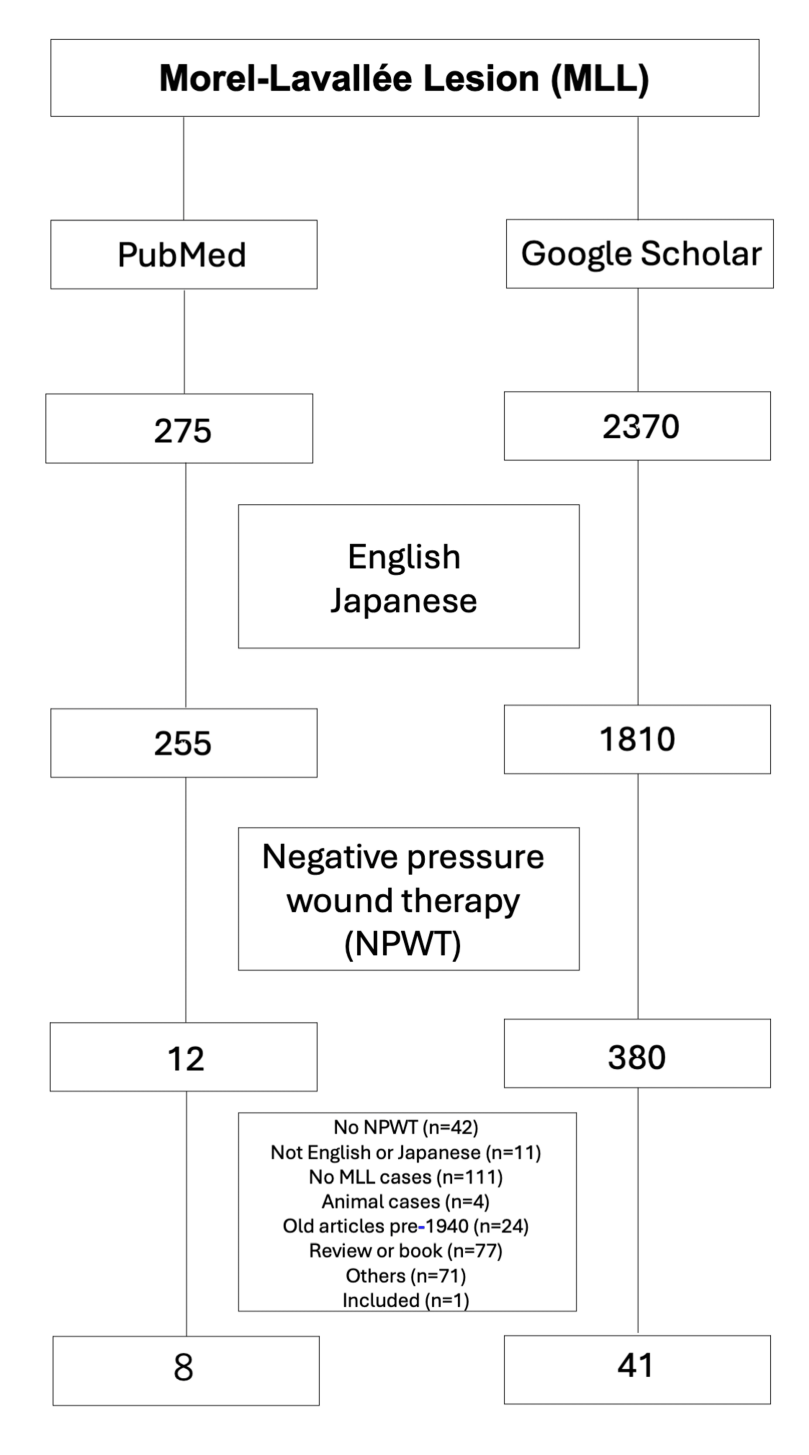
Flow of literature review Two reviewers performed manual screening in a PubMed search and in a Google Scholar search of the English and Japanese literature using the keywords "Morel-Lavallée lesion" and "negative pressure wound therapy". Two reviewers manually screened the remaining titles and abstracts with full texts for relevance. Individual case reports were excluded unless other approaches besides NPWT were discussed. As a result, 12 full-text articles from the PubMed search and 380 full-text articles from the Google Scholar search were initially downloaded. A review of the reference lists from these articles provided one further article for inclusion. All eight articles from the PubMed search were included among the 41 articles from the Google Scholar search. Finally, 41 articles were included in the study. NPWT: negative pressure wound therapy

We encountered a case of MLL that was diagnosed relatively early and successfully treated with portable NPWT. MLL results from a traumatic, internal closed degloving injury [1-4]. These lesions are easily missed initially and can also be difficult to associate with a specific inciting trauma event. Treatment with portable NPWT was non-invasive and effective in this case. Early diagnosis of MLL is crucial for effective treatment with NPWT. However, due to their location in subcutaneous tissues and lack of immediate symptoms, MLL is often overlooked or misdiagnosed. Prompt identification and treatment are essential to minimize treatment duration. The case highlights the difficulty in accurately diagnosing MLL, even in a hospital setting. To date, there has been no literature review on MLL treated with NPWT. The aim of this article is to report on the effectiveness of NPWT in the treatment of MLL. Only 12 published articles were identified in a PubMed search, but 380 published articles were identified from a Google Scholar search of the English and Japanese literature using the search terms "Morel-Lavallée lesion" and "negative pressure wound therapy". Two reviewers independently screened the remaining titles and abstracts. Full-text articles were retrieved for those deemed potentially relevant. Individual case reports were included unless they did not discuss the use of NPWT. Our inclusion criteria specified cases of MLL in which NPWT was employed at any point during treatment. For case series, we conducted a thorough review to identify cases where NPWT was used, even if it was only a part of the overall treatment regimen. Any case that did not involve NPWT was excluded from the analysis. As a result, 12 full-text articles from the PubMed search and 380 full-text articles from the Google Scholar search were initially downloaded. A review of the reference lists from those articles provided one additional article for inclusion. Finally, 41 articles were included in the study with 96 MLL patients (60 males, 36 females) treated using NPWT. A comprehensive literature search was conducted following the Preferred Reporting Items for Systematic Reviews and Meta-Analyses (PRISMA) guidelines (Figure 5).

Etiology

MLL is a traumatic, internal closed degloving injury that results when the subcutaneous tissue shears away from the underlying fascia, usually in association with high-velocity trauma [1-4]. The most common causes of MLL are high-velocity trauma, crush injury, and blunt force trauma [2]. About 50 out of the 96 cases of MLL resulted from motor vehicle accidents, with falls as the second most common cause of MLL (10 of 96 cases). The shear force that separates the underlying fascia from the subcutaneous tissue results in injury to trans-aponeurotic capillaries and lymphatics, leading to hemolymph accumulating within the newly created space.

Pathophysiology

In patients with no symptoms other than swelling, the diagnosis of MLL may be confused with hematoma, soft tissue edema, or bursitis, delaying diagnosis. Blood within the cavity is reabsorbed, and the hemosiderin-rich hemolymphatic fluid (serosanguineous fluid) remains surrounded by a hemosiderin layer [1]. This layer induces inflammation in peripheral tissues and the development of a fibrous capsule around the hemolymphatic fluid, preventing reabsorption [1]. Cavity infection and skin necrosis have been observed in untreated chronic MLL.

Signs and symptoms

MLL typically presents within hours to days after trauma, but up to one-third of cases present months to years later and may be missed initially or may prove difficult to associate with a specific inciting trauma event [5,6]. Clinical findings resemble those of regional contusion. Patients may experience decreased cutaneous sensation because of shearing injury to the cutaneous afferent nerves and increased mobility of the overlying skin [6]. Secondary changes to the overlying skin can include ecchymosis, edema, cracking, drying, abrasions, frank necrosis, and even the absence of any external signs [6,11]. Some patients present with a recurrence of the soft tissue lesion, particularly after minimally invasive treatments [12]. Although MLL is admittedly rare, persistent subcutaneous fluid collection in the setting of trauma should raise the clinical suspicion of underlying MLL.

Evaluation

Imaging is an important adjunct used for diagnosis when MLL is suspected. Features of MLL on ultrasound (US) are non-specific. MLLs are usually compressible without flow on color Doppler US, displaying a focal heterogeneous appearance with irregular margins and a lobular shape in the acute or subacute stage [4,11,13]. Chronic MLL is better defined, homogeneous, and smoothly marginated on US, located between the subdermal fat and fascia [4,11,13]. CT of MLL has limited value and often demonstrates fluid-fluid levels reflecting the internal content of immiscible lymphatic fluid and hemorrhage. The density of MLL on CT is usually lower than that of simple hematoma because of the mixing of low-density lymphatic fluid. In chronic lesions, a peripheral capsule may be present [4,11,13]. MRI is the preferred imaging modality for the evaluation of MLL. Signal characteristics depend on the internal contents of the lesion. Acute MLL appears hypointense on T1-weighted imaging and hyperintense on T2-weighted imaging. Subacute lesions may appear hyperintense on T1-weighted imaging due to the methemoglobin content. Chronic lesions appear homogeneously hypointense on T1-weighted imaging and hyperintense on T2-weighted imaging. In chronic lesions, a peripheral ring or capsule that appears hypointense on T1- and T2-weighted imaging may be present, representing hemosiderin and fibrous tissue [11].

Management

No specific guidelines are available in the current literature regarding the management of MLL. Several small cohort studies have shown variable results from multiple treatment modalities, including conservative treatment, percutaneous aspiration, sclerotherapy, minimally invasive surgery, open surgery, and NPWT.

Conservative Treatment

Compression bandaging with analgesics has been advocated for small, acute lesions where no capsule is present, but effective compression is difficult in the trochanteric and pelvic regions where MLL is common. In cases of large or chronic MLL, conservative management is unsuitable, and surgical intervention is required.

Percutaneous Aspiration

Some studies have shown effective results after percutaneous aspiration of MLL, but the recurrence rate was high, particularly for lesions with a volume >50 ml and even more so for cases requiring multiple aspirations [5].

Sclerotherapy

This treatment modality has been successfully used in MLL, particularly in cases where percutaneous aspiration fails. Potential sclerosants include doxycycline, erythromycin, vancomycin, tetracycline, bleomycin, absolute ethanol, and talc. Fibrin glue has also been used with satisfactory results [14]. The overall efficacy of sclerotherapy in managing MLL has been reported as 95.7% [12].

Minimally Invasive Surgery

This approach involves the removal of the fibrotic pseudocapsule with the obliteration of any dead space. A minimally invasive method through endoscopy is used to debride the fibrotic pseudocapsule. Various methods can be applied for the obliteration of dead space, including doxycycline, fibrin glue, loop drainage, and percutaneous cutaneo-fascial suture technique [15-18]. NPWT was also applied in some cases with minimally invasive surgery, as described later.

Open Surgery

Most cases of MLL require open debridement with the complete removal of necrotic tissue in acute cases showing overlying skin necrosis and with excision of the pseudocapsule in chronic cases. The end goal in managing MLL is the obliteration of dead space within the lesion. Previous studies have found that the overall recurrence rate was lower in the surgical group than in the percutaneous aspiration group [2,5]. In cases where the skin overlying the lesion is necrotic with massive soft tissue loss, dead tissue needs to be debrided, followed by reconstruction with skin grafts [4]. If open surgery failed, the last treatment would be en bloc resection of the lesion with surrounding intact tissues [12].

NPWT

MLL treated with open surgery management can recur in up to 15% of patients [5]. In the setting of chronic or recurrent MLL, NPWT can offer several benefits regarding dead space management. NPWT applies contracting force on the wound to minimize dead space and create a stable wound environment by draining excessive fluid, in turn supporting the formulation of granulation tissue [19]. The reduction of tissue edema within the wound can enhance cell proliferation and tissue perfusion [19].

Recent studies on 96 MLL patients (60 males, 36 females; average age, 40 years) described NPWT. In these studies, after percutaneous aspiration or incisional debridement combined with vacuum-assisted closure, NPWT was applied. Favorable outcomes were reported for the majority of patients, besides three failure cases and four deaths. No evidence of lesion recurrence or infection was seen, suggesting the effectiveness of this treatment. The three cases of NPWT failure showed healing of the MLL after secondary intention [20-22]. We also found several cases in which healing was achieved with additional debridement after NPWT [20,21,23-27].

The single-use disposable portable NPWT was used in one case for acute sacral MLL [9]. The single-use ultraportable NPWT device delivers negative pressure at -80 mmHg in a continuous pattern and is a small, canister-free device that is easy to operate and less uncomfortable in daily life [28].

Table 1 shows the 41 studies with 96 MLL patients (60 males, 36 females) treated using NPWT.

**Table 1:** Cases treated with NPWT We used those cases in which NPWT was used to treat MLL and thus omitted cases in which MLL was not present or in which MLL was present but NPWT was not used (not all cases covered by Cormican et al. [34], Dodwad et al. [23], George et al. [37], Labler and Trentz [44], Marangi et al. [46], Nakajima et al. [50], Takahara et al. [56], and Watfa et al. [58] were therefore included in this review). The age of Thoppanahalli Venkatesh et al.'s patient was described in his 40s; thus, 45-year-old was used. Failure was defined as the need for open surgery following NPWT. Additional surgery was a minor procedure such as irrigation and debridements. CI: crush injury; F: female; M: male; MLL: Morel-Lavallée lesion; MVA: motor vehicle accident; NPWT: negative pressure wound therapy; STSG: split-thickness skin graft

Author (alphabetical)	Sex	Age	Surgery	Mechanism of injury	Remarks	Failure	Additional surgery
Archer et al. [29]	M	31	Open surgery (debridement surgery)	Industrial sandblast injury	None	None	None
Blome-Eberwein [30]	F	13	Drained via inferior and superior incisions	MVA	None	None	None
Brown et al. [31]	F	30	Open surgery	MVA	Abdominal friction burns (TBSA 13%)	None	None
Bruce et al. [32]	M	46	Open surgery	MVA	None	None	None
Choi et al. [33]	F	53	Open surgery (limited incisional drainage)	MVA	None	None	None
Choi et al. [9]	M	51	Percutaneous aspiration	Fall from a height of 3 m	None	None	None
Cormican et al. [34]	F	61	Open surgery (multiple debridement)	MVA	Only case 2 used NPWT for MLL	None	None
Dodwad et al. [23]	M	58	Open surgery (multiple debridement)	MVA	Only case 3 used NPWT for MLL	None	+
Eldenburg et al. [24]	F	25	Open surgery, STSG	MVA	MSSA infection	None	+
Sönmez Ergün et al. [35]	F	51	CT-guided aspiration, open surgery	MVA	As a pedestrian, the patient was struck by a car	None	None
Evin [36]	16 M, 7 F	Mean 33.3	Open surgery	13 MVA, 10 CI	None	None	None
George et al. [37]	M	41	Surgical debridement, oxytetracycline solution, drainage, and cyst wall excision	MVA	Only case 2 used NPWT for MLL	None	None
Gunay et al. [38]	M	47	Open surgery	MVA	Gram-positive cocci (*Streptococcus* spp.) and Gram-negative rod (*Escherichia coli*), death on hospital day 15 due to septic shock	None	None
Haydon and Zoumaras [39]	F	73	Open surgery	MVA	None	None	None
Hefner et al. [20]	M	28	Open surgery	MVA	Methicillin-resistant *Staphylococcus aureus* infection	Failure	+
Heo and Kim [40]	F	66	Open surgery	MVA	Died of aspiration pneumonia on hospital day 125	None	None
Howell et al. [41]	F	60	Open surgery	Unconscious for an unknown period	Schizophrenia, bipolar disorder, substance use disorders	None	None
Hu et al. [42]	F	49	Open surgery	MVA	None	None	None
Kage et al. [7]	M	51	Minimally invasive surgery, arthroscope	MVA	None	None	None
Kim et al. [43]	M	43	Minimally invasive surgery	MVA	None	None	None
Labler and Trentz [44]	6 M, 2 F	Mean 30.3	Open surgery	10 MVAs, 2 falls from great height, 1 working accident	8 MLL cases in total 13 cases, 18-year-old male died on hospital day 6	None	None
Lee et al. [45]	1 M, 1 F	Mean 46	Open surgery	Amateur goalkeeper with a history of regularly diving for blocking, repetitive sitting position during housework	Uncharacteristic clinical features	None	None
Mahajan et al. [21]	F	25	Percutaneous drainage and compression bandage, open surgery	MVA	None	Failure	+
Marangi et al. [46]	5 M	Mean 51	Open surgery	Uncertain mechanism of injury	5 MLL cases in total 15 cases	None	None
Mettu et al. [47]	F	35	Minimally invasive surgery arthroscopic shaver	MVA	None	None	None
Miura [22]	M	41	Conservative, open surgery	MVA	None	Failure	None
Mooney et al. [48]	F	48	Open surgery	Slip and fall on ice landing directly on lower back 1 month before presentation	None	None	None
Mulcahy and Ball [49]	2 M	Mean 21	Open surgery	2 MVAs	None	None	None
Nakajima et al. [50]	M	26	Open surgery	Legs caught between rollers during work	Only case 2 used NPWT for MLL	None	None
Nica et al. [25]	M	31	Open surgery	MVA	None	None	+
Nicolas et al. [51]	M	45	Open surgery (multiple debridement)	While playing football, collided with another individual, friction burn on synthetic grass	MLL stage VI	None	None
Ning and Zha [10]	4 M, 9 F	Mean 54.2	Open surgery (mesh incision combined with mattress suturing and negative-pressure drainage)	7 MVAs, 6 fall injuries	None	None	None
Ozer et al. [52]	9 M	Mean 24.3	Open surgery	6 land mine explosions, 2 gunshot injuries, 1 MVA	A 32-year-old patient died on hospital day 6	None	None
Park and Kim [53]	M	83	Open surgery	No trauma described	Methicillin-resistant *Staphylococcus aureus* infection	None	None
Phillips et al. [27]	F	41	Open surgery	MVA	*Escherichia coli*, *Klebsiella pneumoniae*, *Enterobacter cloacae*, *Enterococcus faecalis*, diphtheroid *Bacillus* infection	None	+
Row et al. [54]	F	31	Open surgery (5 surgical debridements)	Gunshot	None	None	None
Stiff et al. [55]	F	64	Open surgery excision	Fall 7 years earlier	None	None	None
Takahara et al. [56]	M	65	Aspiration, open surgery, aspiration	MVA	Only case 1 used NPWT for MLL, methicillin-resistant *Staphylococcus aureus* infection	None	None
Thoppanahalli Venkatesh et al. [57]	M	45	Open surgery	MVA (run over by four-wheeler)	None	None	None
Watfa et al. [58]	F	42	Selective lymphatic ligation	Trauma (MLL)	1 MLL case in total 14 cases	None	None
Weiss et al. [26]	M	22	Percutaneous aspiration, open surgery	Fall down stairs	None	None	+

Limitations to this study need to be considered. The effectiveness of treatment using NPWT alone remains unclear. Percutaneous aspiration alone without the use of portable NPWT might also have induced different results (e.g., infection or lesion recurrence). However, we concurrently employed portable NPWT in conjunction with aspiration drainage and minor surgical debridement to promote healing and prevent recurrence.

Nonsurgical treatments such as aspiration drainage are often prioritized in the treatment of MLL. Aspiration of more than 50 mL of fluid from the lesion warrants surgical intervention [5].

NPWT is contraindicated in conditions including malignancy in the wound, untreated osteomyelitis, necrotic tissue with eschar that remains undebrided, nonenteric and unexplored fistulas, exposed organs, exposed vasculature, exposed nerves, and exposed anastomotic site [19].

While the results for the present case were promising, future research needs to expand the number of patients to validate the present findings for NPWT. Future studies would preferably involve a controlled study design, but this may be complicated by the current absence of a "gold standard" for MLL treatment.

## Conclusions

MLL is a traumatic, internal closed degloving injury that results from the subcutaneous tissue shearing away from the underlying fascia. We encountered a patient presenting with MLL who was successfully treated by single-use portable NPWT without invasive treatment. MLL can be treated with portable NPWT when the space is relatively small, the patient is compliant with treatment, the patient is not vasculopathic (e.g., diabetic), and the patient has undergone prior aspiration and debridement.
